# Notes of the Week

**Published:** 1921-07-23

**Authors:** 


### NOTES OF THE WEEK.

### THE B.M.A. AT NEWCASTLE.

Under the presidency of Dr. Garstang, of Altrincham, the representative meeting of the British Medical Association at Newcastle, which commenced on July 16, has debated a number of topical matters. The question of professional secrecy we deal with below. On the second day a series of resolutions were discussed relative to the methods which should be adopted in the conduct of hospitals in the future. The first resolution upheld the necessity of maintaining the voluntary system. One speaker was strong on the point that every citizen should be enabled to obtain hospital treatment should he desire to do so; but he, probably somewhat in advance of his era, did not receive much support. A further motion adopted was on the question of paying patients, and affirmed the principle that fees for professional services should be a matter of arrangement between the patient, the family physician, and the consultant. The meeting adopted a resolution maintaining that the essence of the voluntary system was independent and voluntary management, and that this was in no way related to the conditions of service of the medical staff.

### MEDICAL SECRECY.

The debate at the Newcastle meeting of the British Medical Association on the subject of whether a medical man should be forced to reveal in a Court of Law information imparted to him in confidence by his patient has attracted public attention once more to this old but never stale controversy. The line which the leaders of the B.M.A. took up was not nearly strong enough to please the bulk of the representatives present, who carried an amendment which expresses practically the views put forward in these columns only last week, written, it need scarcely be said, before the British Medical debate had taken place. Admittedly this thorny subject has many sides to it, and opinions are bound to differ about it. The criterion in our judgment is simply the general advantage of the greatest number of the people. This is difficult to assess in figures, and must remain largely a matter of opinion. There is, however, one further thought for consideration. The privilege allowed to the priest and the lawyer is one which advantages only the individual, himself in most cases one who has acted anti-socially: it is to give the individual the fullest opportunity of saving his own soul or his own skin that his advisers in such matters are allowed to keep secret his confidences. In the case of doctor and patient, there are others whose interests are affected. If the patient with venereal disease be hindered from seeking proper treatment by the fear that his condition may be disclosed to third parties, it is not only his own health that may suffer; he (or she) may infect others, including innocent offspring, and the catastrophes thus started may spread far and wide, to the patent detriment of the State, which is therefore deeply concerned in seeing that its laws should be such as really conduce to the welfare of the greatest number.

### THE MISCHIEF IN COCKTAILS.

In the recent press controversy concerning the dangers of cocktail-drinking, one is decidedly more impressed with the medical explanation of its risk than the Americans' defence of its virtues. Actually it is largely a matter of habit, and every daily habit has its staunch defenders, but medically one does not consider its expense or its necessity socially. Doubtless, cocktails produce a craved-for stimulus to many a jaded sensibility, but, nevertheless, what it all amounts to is this: Is it hygienically sound to take considerable quantities of alcohol into an empty stomach? As with most aspects of this alcohol question, it is largely a question of amount. A small quantity of alcohol—a quantity that is rapidly absorbed and metabolised in the system—may be positively beneficial. Taken in excess of the body's power immediately to break it up chemically, it becomes first a poison to the nervous system, and in greater quantity a direct poison to the lining membranes of the stomach itself. Obviously, it is in immoderation with this drink, in alcohol in any form, that the danger lies, and not in the fact that it is an American cocktail "introduced by the war."

### A FINANCIAL RECOVERY AT NORWICH.

A cheering improvement is recorded in the finances of the Norfolk and Norwich Hospital, and the latest quarterly statement shows that this has been maintained. The ward closed in March is now reopened, and the expenses for the first six months of this year have been covered by the income received during this period. Last year the hospital was being run at a loss of £250 a week. A remarkable feature in the recovery is that £6,000 has been raised in six months from entertainments alone, a famous example of organised effort. Still more satisfactory is the receipt of £3,200 as a first instalment of the contributory scheme in connection with the Saturday and Sunday Funds. The board urges both the entertainment campaign and the contributory scheme upon all villages which have not yet joined them. The prevalence of unemployment has probably affected the contributions, which may, therefore, be expected to grow into an important part of the annual income. It is noteworthy that, so far, along with the contributions, patients' payments and church collections have also improved. But the big overdraft still remains. Mr. Charles Larking, Chairman of the Finance Committee, made a good point against the supporters of municipal hospitals when he said that the increase in the rates resulting from such a scheme could be illustrated by the homely fact that immediately the Corporation had taken over the Norwich Waterworks it had increased the water-rate of the hospital from £400 to £1,100. If you want to raise your rates, he said in effect, municipalise your hospital. The power of voluntary effort is shown in this remarkable recovery at Norwich.

### THE HOSPITAL PAGEANT.

The second meeting to consider a Hospital Pageant or Hospital Week was held at the National Hospital, Queen Square, last week, and, considering that the holiday season has commenced, was very well attended. Pressure on our space compels us to hold over the report of the proceedings, which we hope to publish in full next week. It was very clear that neither the Central Fund nor the hospitals care to express very definite views on the subject until the proposals are put in a more concrete form as regards probable cost and the effect to be expected from the effort. The appointment of a representative executive committee, which took place at the meeting, was clearly the first step, and from it should ensue the main lines upon which the Week and Pageant shall be conducted, and, within a little, an estimate of the cost involved. The trend of the debate on July 15 indicated that great immediate financial results are not expected, the scheme being one of an advertising nature on a large and novel, although not necessarily expensive, scale. Already the mickle which makes a muckle is steadily coming from the general public, and if the "hospital idea" can be brought home to each household the battle that the voluntary hospitals are fighting will practically be won.

### A RESULT OF MASS CONTRIBUTIONS.

The Prince of Wales, who was prevented by indisposition from meeting the workers of the League of Mercy at the garden-party at St. James's Palace, on Wednesday, July 13, wrote a letter thanking them for their past successful efforts. He continued: "Now that the voluntary hospital system has been vindicated by our Government Committee I trust that all workers for the League will renew and redouble their efforts on behalf of our voluntary hospitals." The League of Mercy, of which H.R.H. is the Grand President, has distributed in the twenty-two years of its existence no less a sum than £509,000 through the King's Fund to the London hospitals. By far the greater part of this amount has been collected in small subscriptions of under £1, the whole of which is devoted to the benefit of the hospitals. The League of Mercy is one of the organisations invited by the recent King's Fund Conference to co-operate in the organisation of local collections from employees throughout the London area, and its experience should prove highly valuable.

### A ROYAL VISIT.

On Monday last the President, H.R.H. the Duke of Connaught, paid a visit of an hour's duration to the Royal Westminster Ophthalmic Hospital. His Royal Highness was received on his arrival by the Chairman, the Earl of Arran, P.C., K.P., Mr. B. E. H. Bircham, J.P. (Treasurer), Captain Johnson (Secretary), and the Matron (Miss Richards), and was conducted to the board room, where members of the Committee of Management and medical staff were presented. In Swinburne Ward members of the Ladies' Committee were assembled, and were presented to his Royal Highness. The President made a complete inspection of the hospital, visiting the wards, and having a kindly word for each patient. In the operating-theatre Mr. Cruise, C.V.O., F.R.C.S., gave an interesting exhibition with the giant ring-magnet.

### A FIVE YEARS' RECORD.

The Reports of the Joint War Committee and the Joint War Finance Committee of the British Red Cross Society and the Order of St. John of Jerusalem in England on voluntary aid rendered during 1914 to 1919 to the sick and wounded at home and abroad, and to British prisoners of war, have now been published. They are issued by H.M. Stationery Office in a volume of 823 pages, the price of which is 12s. 6d. net. The contents are divided into eight sections: (1) Headquarters work and finance; (2) work abroad; (3) wounded, and missing and prisoners of war; (4) demobilisation and conclusion; (5) appendices, which include some interesting historical documents and a bibliography of selected books and reports relating to Red Cross work in the war; (6) illustrations; (7) maps; and (8) specifications and drawings. The whole is well printed and arranged, and forms a valuable addition to the literature of the war and a wonderful record of voluntary effort. The magnitude of the work is made evident by the fact that over sixteen and a half million pounds in cash and over a million pounds worth of stores were given by the public, and were administered from the Joint Committees' headquarters; the entire cost of administration was less than 8¼d. in the £ on the income, which would not have been possible without the honorary service rendered by the principal workers. We hope to deal with the report at length in a subsequent issue.

### AN INDEX OF CANCER CURES.

There is something very pathetic about Canon Horsley's appeal in a letter to the *Daily News* for a published index giving the true value of all methods by which cancer is, or has ever been, treated. It seems to us unlikely that people who will pin their faith in defiance of medical advice upon quack cures of so serious a disease as cancer are ever likely to take directions from a printed index. In the first place, it is only necessary that they ask, when in doubt, the opinion of a doctor. And even should they be dissatisfied with less than a specialist opinion, then their medical man can immediately obtain all the necessary information from the larger hospitals. As the *Daily News* itself explains, any sufferer in doubt regarding the value of an alleged cure for cancer can always ask his doctor to appeal to the medical staff of the Cancer Hospital, Fulham Road, or of the Cancer Research Department of the Middlesex Hospital, where they will, doubtless, get the best possible and most willing advice.

